# Neutrophil-to-Lymphocyte Ratios Are Closely Associated With the Severity and Course of Non-mild COVID-19

**DOI:** 10.3389/fimmu.2020.02160

**Published:** 2020-09-02

**Authors:** Sen Qun, Yulan Wang, Jun Chen, Xiang Huang, Hui Guo, Zhaohui Lu, Jinquan Wang, Changcheng Zheng, Yan Ma, Yuyou Zhu, Daqing Xia, Yinzhong Wang, Hongliang He, Yong Wang, Mingming Fei, Yihong Yin, Mao Zheng, Yehong Xu, Wei Ge, Fuyong Hu, Jian Zhou

**Affiliations:** ^1^The First Affiliated Hospital of USTC, Division of Life Sciences and Medicine, University of Science and Technology of China, Hefei, China; ^2^Union Hospital Affiliated with Tongji Medical College of Huazhong University of Science and Technology, Wuhan, China; ^3^Department of Neurology, The Affiliated Hospital of Xuzhou Medical University, Xuzhou, China; ^4^School of Public Health, Bengbu Medical College, Bengbu, China

**Keywords:** Neutrophil-to-lymphocyte ratios, inflammation, cytokines, immune damage, severity and course of non-mild COVID-19

## Abstract

**Background:**

Severe acute respiratory syndrome coronavirus 2 (SARS-CoV-2) infection is spreading worldwide. Measuring the prevention and control of the disease has become a matter requiring urgent focus.

**Objective:**

Based on coronavirus disease 2019 (COVID-19) clinical data from Wuhan, we conducted an in-depth analysis to clarify some of the pathological mechanisms of the disease and identify simple measures to predict its severity early on.

**Methods:**

A total of 230 patients with non-mild COVID-19 were recruited, and information on their clinical characteristics, inflammatory cytokines, and T lymphocyte subsets was collected. Risk factors for severity were analyzed by binary logistic regression, and the associations of neutrophil-to-lymphocyte ratios (N/LRs) with illness severity, disease course, CT grading, inflammatory cytokines, and T lymphocyte subsets were evaluated.

**Results:**

Our results showed that the N/LRs were closely related to interleukin (IL)-6 and IL-10 (*P* < 0.001, *P* = 0.024) and to CD3^+^ and CD8^+^ T lymphocytes (*P* < 0.001, *P* = 0.046). In particular, the N/LRs were positively correlated with the severity and course of the disease (*P* = 0.021, *P* < 0.001). Compared to the values at the first test after admission, IL-6 and IL-10 were significantly decreased and increased, respectively, as of the last test before discharge (*P* = 0.006, *P* < 0.001). More importantly, through binary logistic regression, we found that male sex, underlying diseases (such as cardiovascular disease), pulse, and N/LRs were all closely related to the severity of the disease (*P* = 0.004, *P* = 0.012, *P* = 0.013, *P* = 0.028).

**Conclusions:**

As a quick and convenient marker of inflammation, N/LRs may predict the disease course and severity level of non-mild COVID-19; male sex, cardiovascular disease, and pulse are also risk factors for the severity of non-mild COVID-19.

## Introduction

At present, severe acute respiratory syndrome coronavirus 2 (SARS-CoV-2) infection (1) is largely under control in China, but it is still spreading rapidly worldwide, seriously threatening and damaging global health (2). The prevention and control of coronavirus disease 2019 (COVID-19) is currently the most urgent issue facing the globe.

Many studies have confirmed that inflammatory storms are the key pathological basis determining the clinical symptoms, course, and prognosis of COVID-19 (3). According to the diagnosis and treatment protocol for COVID-19 (seventh edition) (4), the pathologic anatomy of patients with COVID-19 revealed that the lungs, spleen, cardiovascular system, liver, kidney, and other organs were markedly infiltrated by a large number of inflammatory cells, which resulted in functional damage and functional failure. Many studies have confirmed that the impairment of immune function directly affects the course and prognosis of COVID-19 (5). By autopsy, a decrease was observed in the numbers of CD4^+^ and CD8^+^ T cells in the spleen and lymph nodes, and denaturation and necrosis of the spleen and lymph nodes were also found, in addition to the proliferation of spleen macrophages. It has been speculated that T lymphocytes and macrophages may also be important targets for COVID-19 (6).

Severe pneumonia and acute respiratory distress syndrome are the worst outcomes in patients with COVID-19, resulting in cytokine release syndrome and multiorgan failure; the role of IL-6 in the pathological development of patients with COVID-19 has been a focus of research (7, 8), but the function of IL-6 in the course of the disease is still controversial (9–12). Lymphocytopenia is a common feature of COVID-19 and may be a key factor associated with disease severity and mortality (13). It is critically important to identify at the early stage of the infection those patients who would be prone to developing the most adverse effects. Based on clinical data from Wuhan (which had a severe outbreak of COVID-19 early in the pandemic), we conducted an in-depth analysis to clarify some of the pathological mechanisms of the disease and identify simple measures to predict its severity early on.

## Materials and Methods

### Ethical Approval and Consent to Participate

This study was approved by the Research Ethics Committee of the First Affiliated Hospital of the University of Science and Technology of China (USTC) and was therefore performed in accordance with the ethical standards laid down in the 1964 Declaration of Helsinki and its later amendments. All participants gave their informed consent prior to their inclusion in this study. Consent was obtained directly from each patient or from a family member or other legal guardian. If a patient was considered incapable of giving informed consent, a family member or other legal guardian was contacted to give informed consent on behalf of the patient. All patients or their close relations were informed of the purpose of the study and signed informed consent. The consent procedures were approved by the ethics committee.

### Subjects

We collected the clinical data of all inpatients with COVID-19 in the cancer center of Wuhan Union Hospital from February 2, 2020 to March 17, 2020.

### Inclusion and Exclusion Criteria

The diagnostic criteria for suspected cases of COVID-19 pneumonia included the following (4): (1) Epidemiological history included: (i) pre-onset history of travel or residence in Wuhan or other areas where local cases continue to spread, (ii) fever or respiratory symptoms in patients who were from Wuhan City or other areas with continued local spread and were exposed 14 days before onset, and (iii) a cluster or epidemiological association with COVID-19 infection; (2) the following clinical features were present: (i) fever, (ii) imaging features indicative of pneumonia, and (iii) a normal or decreased total number of white blood cells in the early stage of the disease or a reduced lymphocyte count; or (3) if there was any epidemiological history, a suspected case could be diagnosed in the presence of any two of the listed clinical manifestations.

The inclusion criteria were as follows: (1) The case met two criteria: (i) suspected COVID-19 and (ii) detection of COVID-19 nucleic acids by real-time fluorescent RT-PCR using sputum, pharyngeal swabs, or lower respiratory tract exudate; (2) non-mild severity.

The exclusion criteria were as follows: mild cases were defined according to the National Protocol: “the clinical symptoms were mild, and there was no sign of pneumonia on imaging” (4). Given the state of emergency at the time, only patients with moderate or severe cases were admitted to the cancer center of Wuhan Union Hospital; patients with mild symptoms were admitted to the mobile cabin hospital (14).

Clinical data on 230 patients with COVID-19 pneumonia in the isolation ward of the cancer center of Wuhan Union Hospital who met the above criteria were collected from February 2 to March 17, 2020.

### Clinical Classification of Disease Severity

All confirmed patients were clinically classified according to the “Novel Coronavirus Pneumonia Treatment Scheme of the National Health Commission of the People’s Republic of China (version 7)” at the time of admission (4), as follows: (1) general type: symptoms such as fever and respiratory tract complaints were present, and manifestations of pneumonia could be seen on imaging; (2) serious type: any of the following criteria were met: (1) respiratory distress, respiratory rate (RR) ≥ 30 times/min; (2) resting oxygen saturation ≤ 93%; or (3) arterial partial oxygen pressure (PaO_2_)/oxygen absorption concentration (FiO_2_) ≤ 300 mmHg (1 mmHg = 0.133 kPa); (3) critical type: any of the following criteria were met: (1) respiratory failure and a need for mechanical ventilation, (2) shock, or (3) a combination of factors with other organ failure requiring ICU care.

### Course of Disease and CT Grading

The course of disease was defined as the time (days) between the onset of clinical symptoms of COVID-19 and the time of discharge from the hospital (symptoms recovery). All images were independently read by three senior radiological specialists. The location, shape, number, and size of the abnormalities on chest CTs were carefully observed and recorded. In case of discordant reading, consensus was reached during another reading session. CT grading was defined according to the “Novel Coronavirus Pneumonia Treatment Scheme of the National Health Commission of the People’s Republic of China (version 7)”and Chinese expert consensus (4, 15).

### Testing Methods

#### Real-Time Reverse Transcription Polymerase Chain Reaction Tests

The confirmation of COVID-19 was achieved by real-time reverse transcription polymerase chain reaction (RT-PCR) testing of throat sputum, pharyngeal swabs, and lower respiratory tract exudates of suspected patients. Following the recommendation of the China National Centre for Disease Control, two target genes were targeted as described previously (16), namely, open reading frame 1ab (ORF1ab) and nucleocapsid protein (N), and these genes were simultaneously amplified and tested during the real-time RT-PCR assay. The primers for target 1 (ORF1ab) were forward primer CCCTGTGGGTTTTACACTTAA; reverse primer ACGATTGTGCATCAGCTGA; and probe 5′-FAM-CCGTCTGCGGTATGTGGAAAGGTTATGG-BHQ1-3′. The primers for target 2 (N) were forward primer GGGGAACT TCTCCTGCTAGAAT; reverse primer CAGACATTTTGCT CTCAAGCTG; and probe 5′-FAMTTGCTGCTGCTTGACAG ATT-TAMRA-3′. A cycle threshold value (Ct value) less than 37 was defined as a positive result, and a Ct value exceeding 40 was defined as a negative test.

#### Inflammatory Cytokines and General Laboratory Tests

Venous blood was taken in the emergency department at admission and sent to the laboratory for examination of routine blood parameters and inflammatory cytokines. However, T lymphocyte subsets and some biochemical indicators were obtained by testing fasting blood the morning after admission. Serum inflammatory cytokines were detected by enzyme-linked immunosorbent assays for IL-2, IL-4, IL-6, IL-6, IL-10, and tumor necrosis factor-α (TNF-α). The tests were carried out strictly according to the instructions, and the test kits were purchased from eBioscience Company (EPX650-16500-901).

#### Flow Cytometry Assay

In order to quantify T lymphocyte subsets, 100 μl of whole blood was incubated in 900 μl of Tris-NH4Cl buffer (Thermo Fisher Scientific) at room temperature for 5 min to lyse erythrocytes. After two washes with phosphate-buffered saline (PBS), cells were incubated with CD3-APC, CD4-PerCP, and CD8-FITC antibodies (5 μg/ml each, all from BD Biosciences) for 15 min on ice. After another two washes with PBS, cells were resuspended in 500 μl of PBS. Samples were analyzed on a BD FACS Canto Plus flow cytometer. Among all collected events, single events were gated between forward scatter (FSC)-A and FSC-H. Cell debris was excluded, and intact cells were then gated from single events based on FSC-A and side scatter (SSC). Each cell population was then detected based on antibody staining.

### Statistical Analysis

All statistical analyses were conducted with Statistical Package for the Social Sciences version 16.0 (SPSS, Company, Chicago, IL, United States). All continuous data were tested for normal distributions using the Kolmogorov–Smirnov test. Normally distributed variables were described as the mean ± standard deviation (*M* ± SD), and non-normally distributed variables were presented as the median and the interquartile range [median (IQR)]. Categorical variables were expressed as constituent ratios and percentages in each category. One-way ANOVA or Kruskal–Wallis analysis was applied to continuous variables, and the chi-square test or Fisher’s exact test was applied to categorical variables. Differences in cytokine and T lymphocyte subsets tested twice for each patient were assessed by paired *t*-test or Wilcoxon signed rank test. Correlation analysis was used to determine the relationship between N/LRs and inflammatory cytokines, T lymphocyte subsets, severity of illness, course of disease, or CT grading. N/LRs in different patient condition groups were tested by Kruskal–Wallis analysis and the Jonckheere–Terpstra trend test. Binary logistic regression analysis was used to test the risk factors for the severity level of patients with COVID-19. A *P* value < 0.05 was considered statistically significant.

## Results

(1) Clinical characteristics of patients with COVID-19.

We collected 230 patients with COVID-19; 99 (43%) patients were male, the average age was 62 (16) years, and the total course of disease was 27 (18) days. The distribution of severity categories was as follows: 190 (82.6%) patients had the general type, 35 (15.2%) patients had the serious type, and five (2.2%) patients had the critical type. According to CT grading, 99 (43.0%) patients were in the early stage, nearly half (106 patients, 46.1%) were in the progressive stage, and 25 (10.9%) were in the critical stage. The underlying (comorbid) diseases of the patients were as follows: 10 (4.3%) patients had respiratory system diseases, 51 (22.2%) patients had immune-related diseases, 79 (34.3%) patients had cardiovascular system diseases, 15 (6.5%) patients had liver or kidney diseases, and 127 (55.2%) patients had other comorbidities. The patients’ initial symptoms were as follows: 154 (67.0%) patients had fever, 109 (47.4%) patients had respiratory system symptoms, 45 (19.6%) patients had general symptoms, nine (3.9%) patients had cardiovascular system symptoms, 21 (9.1%) patients had digestive system symptoms, 16 (7.0%) patients had nervous system symptoms, and five (2.2%) patients were asymptomatic. The damage to physiological systems was as follows (Multiple system damage were directly caused by COVID-19): 111 (48.3%) patients had respiratory system damage, 14 (6.1%) patients had cardiovascular system damage, 20 (8.7%) patients had liver function damage, 22 (9.6%) patients had renal function damage, 21 (9.1%) patients had digestive system damage, 16 (7.0%) patients had nervous system damage, and 14 (6.1%) patients had muscle damage. A total of 175 (76.1%) patients had general symptoms. Of the patients, three (1.3%) were asymptomatic. The variables with statistical significance were identified by univariate analysis. These variables were sex (male) (*P* = 0.005), underlying disease (cardiovascular system diseases) (*P* = 0.019), multisystem damage (muscle) (*P* = 0.047), course of disease (*P* = 0.007), pulse (*P* = 0.049), systolic pressure (*P* = 0.044), L (*P* = 0. 014), and N/LRs (*P* = 0. 029) (see Table 1).

**TABLE 1:** Clinical characteristics of patients with COVID-19.

	**Total (*n* = 230)**	**General type (*n* = 190)**	**Serious type (*n* = 35)**	**Critical type (*n* = 5)**	**Statistical test**
Age, *M* ± SD, years	59.8 ± 14.0	59.4 ± 13.4	60.9 ± 16.5	69.8 ± 17.3	*F* = 1.490, *P* = 0.228
Sex (Male/Female)	99/131	73/117	23/12	3/2	χ*^2^* = 9.514, *P* = 0.005
CT grading (early stage/progressive stage/critical stage)	99/106/25	83/88/19	14/17/4	2/1/2	χ*^2^* = 4.254, *P* = 0.338
**Underlying disease, *n* (%)**					
respiratory system diseases	10 (4.3)	9 (4.7)	0 (0.0)	1 (20.0)	χ*^2^* = 4.189, *P* = 0.124
immune-related disease	51 (22.2)	38 (20.0)	11 (31.4)	2 (40.0)	χ*^2^* = 3.529, *P* = 0.181
cardiovascular system diseases	79 (34.3)	58 (30.5)	18 (51.4)	3 (60.0)	χ*^2^* = 7.128, *P* = 0.019
liver or kidney diseases	15 (6.5)	13 (6.8)	1 (2.9)	1 (20.0)	χ*^2^* = 2.548, *P* = 0.310
other	127 (55.2)	112 (58.9)	13 (37.1)	2 (40.0)	χ*^2^* = 6.162, *P* = 0.044
**Initial symptom, *n* (%)**					
fever	154 (67.0)	128 (67.4)	22 (62.9)	4 (80.0)	χ*^2^* = 0.605, *P* = 0.754
respiratory system	109 (47.4)	85 (44.7)	22 (62.9)	2 (40.0)	χ*^2^* = 4.033, *P* = 0.138
general symptoms	45 (19.6)	39 (20.5)	4 (11.4)	2 (40.0)	χ*^2^* = 3.050, *P* = 0.180
cardiovascular system	9 (3.9)	9 (4.7)	0 (0.0)	0 (0.0)	χ*^2^* = 1.419, *P* = 0.477
digestive system	21 (9.1)	17 (8.9)	4 (11.4)	0 (0.0)	χ*^2^* = 0.440, *P* = 0.846
nervous system	16 (7.0)	15 (7.9)	0 (0.0)	1 (20.0)	χ*^2^* = 4.736, *P* = 0.092
asymptomatic	5 (2.2)	5 (2.2)	0 (0.0)	0 (0.0)	χ*^2^* = 0.981, *P* = 1.000
**Multisystem damage, *n* (%)**					
respiratory system	111 (48.3)	87 (45.8)	22 (62.9)	2 (40.0)	χ*^2^* = 3.621, *P* = 0.156
cardiovascular system	14 (6.1)	13 (6.8)	1 (2.9)	0 (0.0)	χ*^2^* = 0.561, *P* = 0.783
liver function	20 (8.7)	15 (7.9)	4 (11.4)	1 (20.0)	χ*^2^* = 2.140, *P* = 0.279
renal function	22 (9.6)	18 (9.5)	4 (11.4)	0 (0.0)	χ*^2^* = 0.333, *P* = 0.853
digestive system	21 (9.1)	17 (8.9)	4 (11.4)	0 (0.0)	χ*^2^* = 0.440, *P* = 0.846
nervous system	16 (7.0)	15 (7.9)	0 (0.0)	1 (20.0)	χ*^2^* = 4.736, *P* = 0.092
muscle	14 (6.1)	10 (5.3)	2 (5.7)	2 (20.0)	χ*^2^* = 6.519, *P* = 0.047
general symptoms	175 (76.1)	146 (76.8)	24 (68.6)	5 (100.0)	χ*^2^* = 2.235, *P* = 0.288
asymptomatic	3 (1.3)	3 (1.6)	0 (0.0)	0 (0.0)	χ*^2^* = 1.208, *P* = 1.000
Course of disease, Median (IQR), days	27 (18)	26 (15)	35 (21)	50 (48)	χ*^2^* = 9.811, *P* = 0.007
Body temperature, *M* ± SD, °C	35.8 ± 0.5, *n* = 93	35.9 ± 0.4, *n* = 72	35.8 ± 0.7, *n* = 18	36.6 ± 0.0, *n* = 3	*t* = -1.393, *P* = 0.178
Pulse, *M* ± SD, times/min	82.4 ± 11.0, *n* = 175	81.6 ± 10.3, *n* = 150	87.9 ± 13.7, *n* = 20	84.6 ± 14.8	*F* = 3.073, *P* = 0.049
Breathe, *M* ± SD, times/min	20.2 ± 1.9, *n* = 174	19.9 ± 1.1, *n* = 149	21.9 ± 4.3, *n* = 20	20.6 ± 1.3	*Z* = 4.919, *P* = 0.085
Systolic pressure, *M* ± SD, mmHg	128.5 ± 20.8, *n* = 169	127.5 ± 20.3, *n* = 144	130.1 ± 21.3, *n* = 20	150.8 ± 24.6	*F* = 3.173, *P* = 0.044
Diastolic pressure, *M* ± SD, mmHg	78.7 ± 11.1, *n* = 169	78.4 ± 11.4, *n* = 144	79.4 ± 9.3, *n* = 20	85.6 ± 8.4	*F* = 1.045, *P* = 0.354
IL-2, Median (IQR), pg/ml	2.52 (0.68), *n* = 216	2.52 (0.71), *n* = 180	2.47 (0.60), *n* = 32	2.72 (1.69), *n* = 4	*Z* = 1.263, *P* = 0.532
IL-4, Median (IQR), pg/ml	2.03 (1.16), *n* = 215	2.03 (1.18), *n* = 179	1.98 (0.75), *n* = 32	2.81 (2.30), *n* = 4	*Z* = 2.322, *P* = 0.313
IL-6, Median (IQR), pg/ml	16.70 (61.88), *n* = 216	14.69 (61.50), *n* = 180	27.70 (83.96), *n* = 32	22.41 (33.37), *n* = 4	*Z* = 1.035, *P* = 0.596
IL-10, Median (IQR), pg/ml	3.00 (1.87), *n* = 216	3.00 (1.78), *n* = 180	2.91 (1.94), *n* = 32	5.23 (3.36), *n* = 4	*Z* = 5.854, *P* = 0.054
TNF-α, Median (IQR), pg/ml	3.10 (2.52), *n* = 216	3.10 (2.51), *n* = 180	3.08 (2.33), *n* = 32	3.23 (4.74), *n* = 4	*Z* = 0.553, *P* = 0.758
INF-γ, Median (IQR), pg/ml	1.96 (1.25), *n* = 216	1.96 (1.19), *n* = 180	1.90 (1.53), *n* = 32	2.94 (5.05), *n* = 4	*Z* = 2.009, *P* = 0.366
IL-6/IL-10	5.32 (17.59), *n* = 216	4.76 (16.67), n = 180	7.01 (20.88), n = 32	4.96 (4.09), n = 4	*Z* = 1.653, *P* = 0.438
CD3^+^ (%)	76.22 (12.62), *n* = 217	76.49 (11.53), *n* = 182	75.57 (15.20), *n* = 32	68.52 (15.17), *n* = 3	*Z* = 2.250, *P* = 0. 325
CD4^+^ (%)	46.29 ± 10.61, *n* = 218	46.38 ± 10.52, *n* = 183	45.43 ± 11.64, *n* = 32	49.74 ± 4.03, *n* = 3	*F* = 0.270, *P* = 0. 764
CD8^+^ (%)	24.56 ± 9.56, *n* = 218	23.08 (11.39), *n* = 183	21.14 (14.78), *n* = 32	14.47 (9.75), *n* = 3	*Z* = 3.660, *P* = 0. 160
CD4^+^/CD8^+^	2.02 (1.26), *n* = 218	1.98 (1.26), *n* = 183	2.33 (1.21), *n* = 32	3.63 (1.35), *n* = 3	*Z* = 3.605, *P* = 0. 165
WBC (10^9^/l)	5.36 (2.31)	5.32 (2.22)	5.40 (2.54)	6.72 (10.32)	*Z* = 2.480, *P* = 0. 289
N (10^9^/l)	3.23 (1.81)	3.23 (1.68)	3.10 (1.92)	4.32 (10.02)	*Z* = 3.799, *P* = 0. 150
L (10^9^/l)	1.38 ± 0.50	1.42 ± 0.49	1.20 ± 0.48	1.03 ± 0.44	*F* = 4.319, *P* = 0. 014
M (10^9^/l)	0.51 (0.30), *n* = 148	0.52 (0.30), *n* = 112	0.49 (0.30), *n* = 33	0.70 (0.31), *n* = 3	*Z* = 1.951, *P* = 0. 377
N/LRs	2.44 (1.52)	2.41 (1.39)	2.96 (2.47)	6.32 (15.52)	*Z* = 7.094, *P* = 0. 029

*Values of cytokines, T lymphocyte subsets, and classification of leukocytes were the results of the first test after admission. If any values were missing, the sample size was stated. IL, interleukin; TNF, tumour necrosis factor; IFN, interferon; WBC, white blood cells; N, neutrophil; L, lymphocyte; M, monocyte; N/LR, neutrophil/lymphocyte ratio.*

(2) There were significant differences between the first test and last test regarding the cytokines and classification of leukocytes.

We collected the data regarding the cytokines and T lymphocyte subsets and classification of leukocytes at the time of admission (first test) and the time of discharge (last test), and there were statistically significant differences in cytokine levels between admission and discharge. For example, IL-6 and IL-10 were significantly decreased and increased, respectively (*P* = 0.006, *P* < 0.001). The IL-6/IL-10 ratio was also significantly different (*P* < 0.001). Additionally, there were statistically significant differences in lymphocytes and N/LRs between admission and discharge (*P* = 0.002, *P* < 0.001). However, there were no statistically significant differences in T lymphocyte subsets between admission and discharge (Table 2).

**TABLE 2:** Cytokines, T lymphocyte subsets, and classification of leukocytes in patients with COVID-19 (*N* = 230).

**Variable**	**First test**	**Last test**	**Statistical test**
IL-2 (pg/ml)	2.52 (0.68) (*n* = 216)	3.68 (1.17) (*n* = 84)	*Z* = −7.044, *P* < 0.001 (*n* = 81)
IL-4 (pg/ml)	2.03 (1.16) (*n* = 215)	3.56 (1.47) (*n* = 84)	*Z* = −6.901, *P* < 0.001 (*n* = 81)
IL-6 (pg/ml)	16.70 (61.88) (*n* = 216)	9.82 (35.96) (*n* = 84)	*Z* = −2.744, *P* = 0.006 (*n* = 81)
IL-10 (pg/ml)	3.00 (1.87) (*n* = 216)	4.79 (2.20) (*n* = 82)	*Z* = −5.904, *P* < 0.001 (*n* = 79)
TNF-α (pg/ml)	3.10 (2.52) (*n* = 216)	4.40 (4.43) (*n* = 84)	*Z* = −5.172, *P* < 0.001 (*n* = 81)
INF-γ (pg/ml)	1.96 (1.25) (*n* = 216)	3.45 (1.30) (*n* = 81)	*Z* = −5.811, *P* < 0.001 (*n* = 78)
CD3^+^ (%)	76.22 (12.62) (*n* = 217)	76.55 ± 10.09 (*n* = 35)	*Z* = −0.068, *P* = 0.946 (*n* = 31)
CD4^+^ (%)	46.29 ± 10.61 (*n* = 218)	46.10 ± 12.53 (*n* = 35)	*t* = 1.404, *P* = 0.171 (*n* = 31)
CD8^+^ (%)	24.56 ± 9.56 (*n* = 218)	23.65 ± 7.97 (*n* = 35)	*t* = 0.711, *P* = 0.483 (*n* = 31)
CD4^+^/CD8^+^	2.02 (1.26) (*n* = 218)	2.27 ± 1.08 (*n* = 35)	*Z* = −0.843, *P* = 0.399 (*n* = 31)
WBC (10^9^/l)	5.36 (2.31) (*n* = 230)	5.65 ± 1.52 (*n* = 79)	*Z* = −0.237, *P* = 0.813 (*n* = 79)
N (10^9^/l)	3.23 (1.81) (*n* = 230)	3.01 (1.66) (*n* = 78)	*Z* = −1.594, *P* = 0.111 (*n* = 78)
L (10^9^/l)	1.38 ± 0.50 (*n* = 230)	1.58 (0.63) (*n* = 78)	*Z* = −3.131, *P* = 0.002 (*n* = 78)
M (10^9^/l)	0.51 (0.30) (*n* = 148)	0.49 (0.21) (*n* = 78)	*Z* = −1.432, *P* = 0.152 (*n* = 78)
N/LRs	2.44 (1.52) (*n* = 230)	1.92 (1.09) (*n* = 78)	*Z* = −3.813, *P* < 0.001 (*n* = 78)
IL-6/lL-10	5.32 (17.59) (*n* = 216)	2.01 (8.64) (*n* = 82)	*Z* = −4.272, *P* < 0.001 (*n* = 79)

(3) Risk factors for the severity of COVID-19.

The risk factors for the severity of COVID-19 were analyzed by binary logistic regression. Due to the small number of critical-type samples, the critical type and the serious type were grouped together. Variables with statistically significant differences among different groups of diseases by univariate analysis were substituted into the regression equation, and finally, four variables with statistical significance were selected: male sex, underlying disease (cardiovascular disease), pulse, and N/LRs were all closely related to the severity of the disease (*P* = 0.004, *P* = 0.012, *P* = 0.013, *P* = 0.028) (Table 3).

**TABLE 3:** Risk factors for the severity of COVID-19 by binary logistic regression analysis.

	**OR (95% CI)**	***P* value**
Sex (male)	0.189 (0.060–0.589)	0.004
Underlying disease (cardiovascular system diseases)	4.028 (1.360–11.934)	0.012
Pulse	1.055 (1.011–1.101)	0.013
N/LR	1.196 (1.020–1.403)	0.028

*Forward stepwise selection (LR).*

(4) N/LRs are closely related to severity level and disease course.

We found that N/LRs were closely related to the severity level and disease course of COVID-19 according to the Kruskal–Wallis test, *P* = 0.021 and *P* < 0.001, respectively (Table 4).

**TABLE 4:** The relationship between N/LRs and the severity of illness, course of disease, and CT grading (*N* = 230).

	**Severity level**	**Disease course**	**CT grading**
N/LR	0.152	0.852	0.07
*P* value	0.021	<0.001	0.292

(5) Patients with higher N/LRs had higher severity and a longer disease course.

**FIGURE 1:**
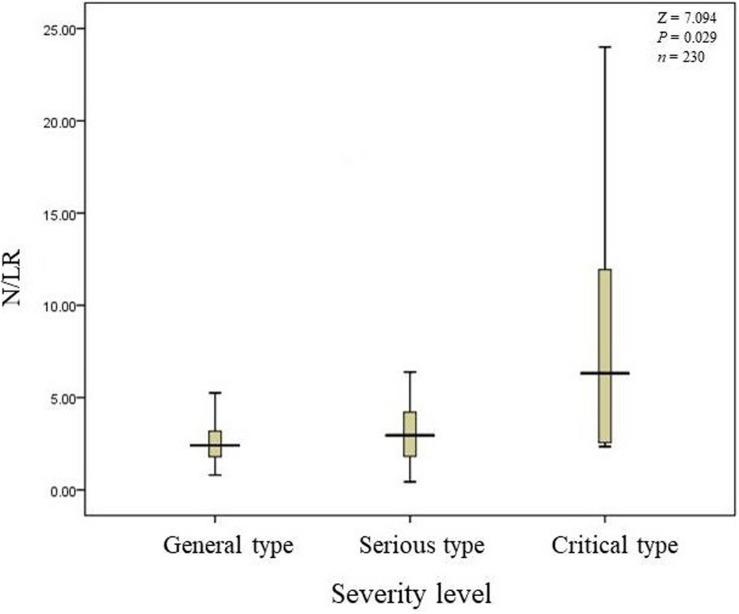
N/LRs in different patient conditions.

**FIGURE 2:**
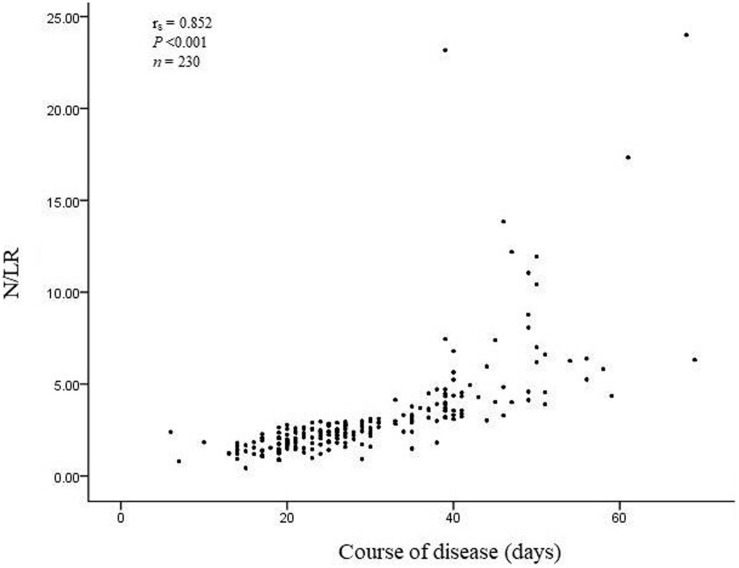
N/LRs in patients with different disease courses.

The total number of COVID-19 patients was 230. 190 patients’ N/LRs were 2.41 (1.39), with general symptoms; 35 patients’ N/LRs were 2.96 (2.47), with serious symptoms; and five patients’ N/LRs were 6.32 (15.52), with critical symptoms, according to the Kruskal-Wallis test (*Z* = 7.094, *P* = 0.029) (Table 1). The difference between each pair of groups was statistically significant. Patients with higher N/LRs had more severe disease. A Jonckheere-Terpstra trend test was performed and showed a statistically significant difference between each group; the more severe the disease, the higher the N/LR (Figure 1). Patients with higher N/LRs had longer disease courses (Figure 2) (*P* < 0.001).

(6) N/LRs were closely related to IL-6 and IL-10.

**FIGURE 3:**
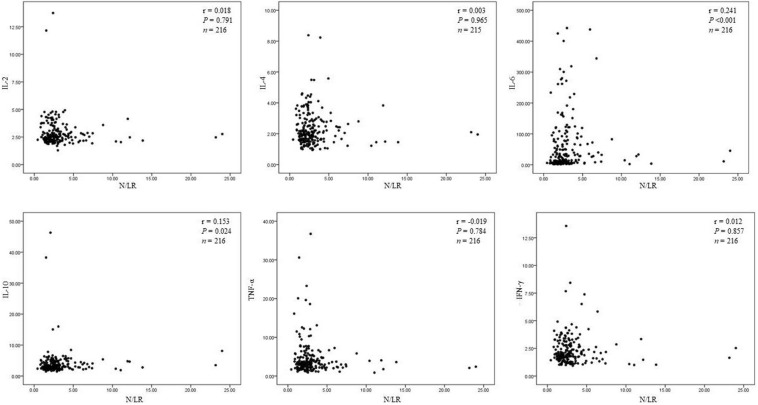
The relationship between N/LRs and different inflammatory cytokines.

We found that N/LRs were positively related to IL-6 and IL-10, *P* < 0.001 and *P* = 0.024, respectively. However, there was no significant correlation with other cytokines (Figure 3). The trend of the relationship between N/LRs and different inflammatory cytokines is shown in Figure 3.

(7) N/LRs are closely related to CD3^+^ and CD8^+^ cells (T lymphocyte subsets).

**FIGURE 4:**
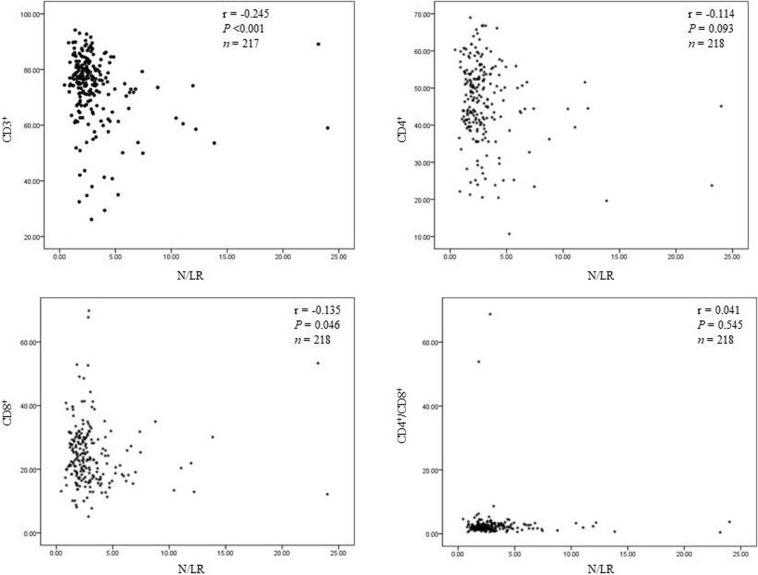
The relationship between N/LRs and different T lymphocyte subsets.

We found that N/LRs were negatively related to CD3 ^+^ and CD8 ^+^ T lymphocytes (*P* < 0.001 and *P* = 0.046, respectively; Figure 4). The trend of the relationship between N/LRs and different T lymphocyte subsets is shown in Figure 4.

## Discussion

In this study, we analyzed the clinical data of 230 patients diagnosed with non-mild COVID-19 in the cancer center of Wuhan Union Hospital and summarized the clinical characteristics of this disease. We found the following: (1) N/LRs, male sex, underlying disease (cardiovascular disease), and pulse were all closely related to the severity of the disease; (2) N/LRs were positively correlated with the levels of cytokines (IL-6, IL-10); (3) N/LRs were negatively correlated with the proportion of CD3^+^ and CD8^+^ T lymphocyte subsets; (4) N/LRs were positively correlated with the severity of the disease, as the higher N/LRs were, the more serious the disease; (5) N/LRs were related to the course of disease, as the higher N/LRs were, the longer the course of disease; and (6) most patients had multisystem symptoms and injury and had underlying multisystem comorbidities.

As the COVID-19 epidemic continues to spread across the globe, effective treatment is urgent, and it is particularly crucial to explore the pathogenesis of the disease. COVID-19 infection activates innate and adaptive immune responses. However, uncontrolled damage from the natural inflammatory response and adaptive immune response may lead to local and systemic tissue damage. Lymphocytopenia is a common feature in patients with severe COVID-19, with a dramatic decrease in the number of CD4^+^ and CD8^+^ T cells, B cells, and natural killer (NK) cells (5, 17–19) and lower proportions of monocytes, eosinophils, and basophils among leukocytes (18, 20); in particular, patients with higher N/LRs have poorer outcomes (20). Yang et al. found that NLRs can be considered independent biomarkers for indicating poor clinical outcomes (21). Consistent with the above data, our research found that the NLRs were closely related to the severity and course of patients with non-mild COVID-19 and that higher N/LRs were related to longer courses and more severe non-mild COVID-19.

Currently, N/LRs are a well-known marker of systemic inflammation and infection, and they have been studied as predictors of bacterial infection, showing superior predictive value over conventional inflammatory markers (22–24). In addition, the N/LRs have displayed good predictive power for pneumonia, as well as dose-response information relating to the burden of community-acquired pneumonia, such as pneumonia severity or mortality (25, 26). Neutrophilia and lymphocytopenia are physiological responses of the innate immune system to systemic inflammation. Lymphocytopenia consists of accelerated apoptosis and margination of lymphocytes within the reticuloendothelial system, liver, and splanchnic lymphatic system and of the redistribution of lymphocytes within the lymphatic system (27–29). As immune changes and inflammation are the core pathological basis of COVID-19, compared with imaging and biochemical examination, N/LRs may be a simpler and more specific way to determine the prognosis of COVID-19.

Most patients with severe COVID-19 show significantly elevated serum levels of inflammatory cytokines, such as IL-6 and IL-1, as well as IL-2, IL-17, G-GSF, GM-GSF, IP-10, MCP1, MIP-1a (also known as CCL3), and TNF, known as a cytokine storm (5, 17–19). In our research, we found that cytokines, such as serum IL-2, IL-4, IL-6, IL-10, TNF-α, and INF-γ, were significantly elevated at admission, and in some patients, these inflammatory cytokines were reviewed before discharge. There were statistically significant differences in cytokine levels at admission and discharge. Interestingly, we found that in the process of treatment, the levels of some inflammatory cytokines were decreased, while others were increased. This phenomenon may be because some inflammatory cytokines are pro-inflammatory and others are anti-inflammatory; these changes may reflect the dynamic transformation process of inflammation from damage to repair during homeostasis and inflammation (30). More importantly, correlation analysis indicated that N/LRs were closely related to IL-6 and IL-10. A recent study showed that M1 macrophages produce pro-inflammatory cytokines, such as IL-6, and that M2 macrophages produce anti-inflammatory cytokines, such as IL-10 (30). Our research found that, compared to those at the first test, IL-10 significantly increased and IL-6 significantly decreased at the last test (*P* < 0.001), which described the patient’s transition from the acute phase to the convalescent phase, and these phenomena may indicate that N/LRs were closely related to injury in the acute stage and repair in the recovery stage in patients with COVID-19. The source, function, and interaction of various inflammatory cytokines are complex. Severe acute respiratory syndrome coronavirus 2-infected macrophages demonstrate upregulation of IL-6 production and low expression of interferons (31, 32). Th1 cell-derived IFN-γ is essential for an effective antiviral immune response. However, IL-6 may inhibit Th1 polarization by stimulating CD4 ^+^ cells to differentiate into Th2 cells or by suppressing IFN-γ expression (33, 34). However, IL-6 can cooperate with transforming growth factor (TGF)-β to induce IL-10 production in Th17 cells (35); IL-10 is also produced by regulatory T cells (Tregs), and TGF-β is critical to enable human Tregs to express IL-10 (36). IL-10 can stimulate lymphocytes, leading to a decrease in the secretion of IL-2 and IFN-γ by T cells (37). Studying the complex inflammatory cytokine network after COVID-19 infection is of great significance for the treatment and prediction of the disease.

Most patients with COVID-19 exhibit mild to moderate symptoms, but approximately 15% progress to severe pneumonia, and approximately 5% eventually develop acute respiratory distress syndrome (ARDS), septic shock, and/or multiple organ failure (5, 17). Wei’s group study showed that after COVID-19 infection, CD4^+^ T lymphocytes are rapidly activated to become pathogenic T helper (Th)1 cells and generate GM-CSF. The cytokine environment induces inflammatory CD14^+^CD16^+^ monocytes with high expression of IL-6 and accelerates inflammation (3). These aberrant pathogenic Th1 cells and inflammatory monocytes may enter the pulmonary circulation in large numbers and play an immune damaging role to cause lung functional disability and quick mortality (5, 38). Their team launched a clinical trial using tocilizumab to block inflammatory storms (IL-6 receptor inhibitor) in severely ill patients and achieved some results (3). Partly corresponding to Wei’s team (3), our clinical study found that the serum level of IL-6 decreased significantly before discharge, which may reflect the pro-inflammatory role of IL-6 in the course of COVID-19 from another perspective. However, our team did not find that inflammatory cytokines (such as IL-6) were significantly different among different severity level groups. Part of the explanation is that there are no mild cases in this study (see section “Materials and Methods”). More importantly, although the role of IL-6 in the pathological development of COVID-19 has attracted much attention (3), its specific role remains controversial (9–12), and several experimental models of viral lung infections suggest that IL-6 demonstrates either pathogenic (39)or protective (40) effects *in vivo*; the consequences of IL-6 induction in COVID-19 may vary depending on the stage of infection and the immune status of the host. The role of these cytokines in SARS-CoV-2 infection should be carefully evaluated.

Severe acute respiratory syndrome coronavirus 2 might act mainly on lymphocytes, especially T lymphocytes (18), and decreases in the levels of CD3^+^ and CD4^+^ T lymphocytes are associated with immunosuppression (41). Our research found that N/LRs were negatively related to CD3^+^ and CD8^+^ T lymphocyte levels, suggesting that the elevated N/LRs reflect the degree of lymphatic impairment, which may support the hypothesis that N/LRs are a sensitive and simple biomarker of immune function in patients with COVID-19.

Additionally, our results suggest that male sex, underlying disease (cardiovascular disease), and pulse are risk factors for COVID-19. The risk associated with male sex raises a topic of great interest: the effects of estrogens on IL-6 and on COVID-19 progression. Estrogens can suppress lipopolysaccharide (LPS)-mediated IL-6 expression in mouse macrophages by both blocking nuclear factor kappa B (NF-kB) activation (42, 43) and inhibiting p38 mitogen-activated protein kinase (MAPK) phosphorylation (43) by acting on estrogen receptors to decrease the production of pro-inflammatory cytokines (42). However, the immunomodulatory effects of estrogens in COVID-19 require further study. Cardiovascular disease and pulse are risk factors for COVID-19, which may suggest that cardiovascular disease has a critical impact on COVID-19 patient mortality (44) and requires more attention as a comorbidity. We found that there were statistically significant differences in cytokines and N/LRs at admission and discharge, while there were no differences in T lymphocyte subsets (CD3^+^/CD4^+^/CD8^+^) in the research. We wanted to know whether the indicators of lymphocyte subset recovery lagged relatively over time and what their significance was. Further work is needed to understand the specific characteristics of the various inflammatory cytokines and T cell subsets in COVID-19, the specific relationship between N/LRs and different inflammatory cytokines and T cell subsets in COVID-19, and the prognostic value for the disease, especially during immunotherapy.

## Conclusion

Post-COVID-19 inflammation is a very complex network system that has a decisive influence on the prognosis of patients. Some inflammatory cytokines (such as IL-6), whose mechanisms and effects are still controversial, need further study. As a quick and convenient marker of inflammation, N/LRs may predict the disease course and severity level of non-mild COVID-19; male sex, cardiovascular disease, and pulse are also risk factors for the severity of non-mild COVID-19.

## Limitations

First, the sample size of critical type patients was relatively small, which may have influenced the results. Second, due to the emergency situation of the epidemic outbreak, there is a certain lack of clinical data, including inflammatory cytokines. We are also collating clinical data from other centers and investigating the mechanisms of inflammatory factors in animal models.

## Data Availability Statement

The raw data supporting the conclusions of this article will be made available by the authors, without undue reservation.

## Author Contributions

SQ was involved in the study design, data interpretation, and manuscript writing and was a recipient of the obtained funding. JZ participated in the analysis, interpretation, and collection of the data. FH participated in the study design and the statistical analysis. ZL, JW, JC, HG, CZ, YM, YZ, DX, YiW, HH, YoW, MF, YY, MZ, and YX were involved in the data collection. YuW and JC participated in the data analysis and data collection. XH participated in the data analysis. WG participated in the data analysis and manuscript writing. All authors contributed to the article and approved the submitted version.

## Conflict of Interest

The authors declare that the research was conducted in the absence of any commercial or financial relationships that could be construed as a potential conflict of interest.
